# Ruptured sinus of valsalva aneurysm presenting as syncope and hypotension: a case report

**DOI:** 10.1186/s12872-021-02247-4

**Published:** 2021-09-17

**Authors:** Guang Ying Zhuo, Pei Yong Zhang, Li Luo, Qian Tang, Tao Xiang

**Affiliations:** Department of Emergency, The Third People’s Hospital of Chengdu, No. 82, Qing Long Street, Chengdu, 610031 Sichuan China

**Keywords:** Sinus of valsalva aneurysm, Acute myocardial infarction, Shock, Syncope, Case report

## Abstract

**Background:**

Unruptured sinus of valsalva aneurysm (SOVA) are typically asymptomatic, and hence can be easily ignored. Ruptured sinus of valsalva aneurysm (RSOVA) usually protrude into the right atrium or ventricular. However, in this case, the RSOVA protruded into the space between the right atrium and the visceral pericardium leading to compression of the right proximal coronary artery. Very few such cases have been reported till date.

**Case presentation:**

We describe a case of ruptured right SOVA in a 61-year-old man with syncope and persistent hypotension. At the beginning, considered the markedly elevated troponin, acute myocardial infarction was considered. However, emergency coronary angiography unexpectedly revealed a large external mass compressed right coronary artery (RCA) resulting in severe proximal stenosis. Then, aorta computed tomography angiography (CTA) and urgent surgery confirmed that the ruptured right SOVA led to external compression of the right proximal coronary artery. Finally, ruptured right SOVA repair and RCA reconstruction were successfully performed, and the patient was discharged with no residual symptoms.

**Conclusions:**

It is very important to be vigilant about the existence of SOVA. RSOVA should be suspected in a patient presenting with acute hemodynamic compromise, and echocardiography should be immediately performed. Moreover, it is very important to achieve dynamic monitoring by using cardiac color ultrasound. Definitive diagnosis often requires cardiac catheterization, and an aortogram should be performed unless endocarditis is suspected.

## Background

Sinus of valsalva aneurysm (SOVA) is usually a congenital anomaly in which a dilatation of the aortic wall is located between the aortic valve and the sinotubular junction. It is rare, estimated at 0.09% of the population, and while they do not always rupture, which usually remains undetected until rupture [1]. SOVA is found most often in the right coronary sinus (RCS), less often in the noncoronary sinus (NCS), and least often in the left coronary sinus. Once ruptured, SOVA can protrude into any of the heart chambers, usually the right atrium or the right ventricle. Occasionally, it ruptures into the pulmonary artery or interventricular septum [2]. Ruptured sinus of valsalva aneurysm (RSOVA) is a rare but well-recognized clinical entity, with a higher incidence in oriental patients than in western populations [3]. Smaller ruptures tend to have a slower onset of symptoms and may be found incidentally, however, it can be catastrophic with significant hemodynamic effects and various symptoms [1].

In this case, we describe the RSOVA protruded into the space between the right atrium and the visceral pericardium, resulting to compression of the right proximal coronary artery. Then, acute myocardial infarction and shock were developed. Very few such cases have been reported till date. Transthoracic echocardiography is the first-line diagnostic tool for this lesion, however, the sensitivity and accuracy of echocardiography is limited, especially in an emergency. In this case, only a small amount of pericardial effusion was found in emergency transthoracic echocardiography. Emergency coronary angiography showed severe proximal right coronary artery (RCA) stenosis, which was related to the external compression of a large mass. Aorta computed tomography angiography (CTA) and urgent surgery confirmed that the ruptured right SVA led to external compression of the right proximal coronary artery. Finally, ruptured right SOVA repair and RCA reconstruction were successfully performed, and the patient was discharged with no residual symptoms.

## Case description

A 61-year-old man was transferred to the emergency department because of fainting. He did not present with any prodromal symptoms before this catastrophic event. Approximately two hours before admission, the patient fainted and was found unresponsive in his bathroom. He regained consciousness after about 30 min. He was admitted to the hospital with persistent confusion, weakness, nausea, dry retching, and cold diaphoresis. His arms and legs were cold and clammy, with no edema. Distal pulse was weak. On arrival at the hospital, additional medical history was obtained from his wife. He had a previous smoking history of one pack/day, but no alcohol or illicit drug use history. His father had hypertension, while his mother had diabetes mellitus. There was no other significant medical, surgical, or family history of any cardiovascular disease. In the emergency department, the systolic blood pressure was 67–70 mm Hg, the heart rate was 106 beats/minute, the respiratory rate was 22 breaths/minute, and the oxygen saturation was 98%, while he was breathing 3 l of oxygen through a nasal cannula. A 12-lead electrocardiogram (ECG) performed within 10 min of arrival was normal. Bedside troponin I level was ≤ 0.05 ng/ml (normal range 0–0.4). Three hours later, the laboratory high-sensitivity cardiac troponin (hs-cTn) T was elevated, at 104.500 pg/ml (normal range 0–14), and six hours later, it had increased to 841.100 pg/ml. The repeat ECG showed atypical non-ischemic changes. Other test results are shown in Table 1.

**Table 1:** Laboratory data

Variable	Reference range, adults	On arrival, emergency department	Pre-operation	Surgery	Day 1, after surgery	Day3, after surgery	Day 3, before discharge
Hemoglobin (g/l)	130–175	125	129	91	68	106	98
Hematocrit (%)	40–50	37.4	39.8	27.8	20.7	32.2	29.4
White-cell count (per mm^3^)	3500–9500	13,100	29,020	24,570	14,620	19,860	11,660
Platelet count (per mm^3^)	125,000–350,000	227,000	118,000	125,000	74,000	59,000	407,000
Prothrombin time (sec)	11.0–15.0	12.7	20.2	22.2	18.5	15.2	14.2
Activated partial-thromboplastin time (sec)	24.0–43.0	33.5	54.5	62.1	55.6	38.1	
D-dimer quantitative detection (mg/l)	0–0.55	0.85	4.12	2.25	3.58	6.14	
Prothrombin-time international normalized ratio	0.8–1.2	0.98	1.76		1.58	1.23	1.10
Potassium (mmol/l)	3.5–5.3	3.81	5.37		4.97	4.21	5.34
Sodium (mmol/l)	135–148	136.5	140.9		146.9	143.9	135.6
Chloride (mmol/l)	96–108	106.0	113.8		108.9	108.7	104.7
Glucose (mmol/l)	3.9–6.1	17.12	6.38		9.75	9.57	
Carbon dioxide (mmol/l)	20–30	14.7	13.5				
Urea nitrogen (mmol/l)	2.9–8.2	5.61	7.95		22.21	23.10	28.04
Creatinine (umol/l)	35–104	120.9	219.8		429.8	350.1	185.5
Alanine aminotransferase (U/l)	0–40	88.5	1874.1		> 2000	1360.3	38.8
Aspartate aminotransferase (U/l)	0–40	145.8	2658.0		> 1500	756.1	28.4
Lactate dehydrogenase (U/l)	109–245	702.3	3325.2		7855	2822.8	
Troponin T (pg/ml)	0–14	104.500	841.100				
Creatine kinase (U/l)	24–195		799.1	537.4		494.7	
Creatine kinase MB isoenzyme (U/l)	0–24	35.48	170.8	102.5		14.4	
Lactic acid (mmol/l)	0.7–2.1	6.97				1.79	
Procalcitonin (ng/ml)	0–0.05	0.06					
B-type natriuretic peptide (pg/ml)	0.00–100.00	24.6		99	521.5	579.5	
*Arterial blood gas*							
Fraction of inspired oxygen		1				0.5	
pH	7.35–7.45	7.35				7.43	
Partial pressure of carbon dioxide (mm Hg)	35–45	25.4				24.1	
Partial pressure of oxygen (mm Hg)	80–100	136.1				139.9	

Reference values are affected by many variables, including the patient population and the laboratory methods used. The ranges used at the Massachusetts General Hospital are for adults who are not pregnant and do not have medical conditions that could affect the results. They may therefore not be appropriate for all patients

**Fig. 1:**
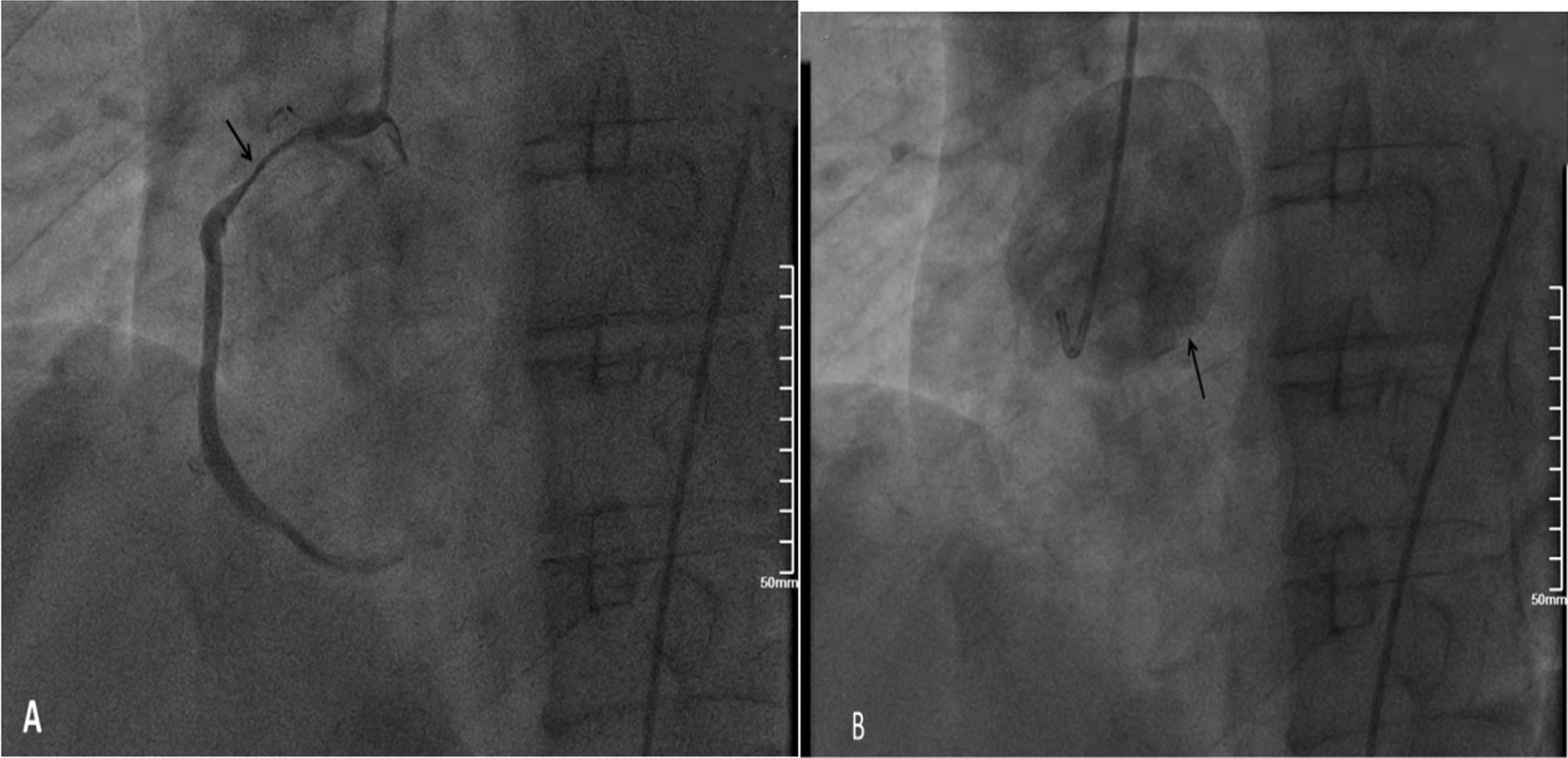
Coronary angiography. Coronary angiography was performed on the patient nine hours after arriving at the emergency department. Coronary angiography showed severe stenosis of the proximal RCA (Panel **A**, arrow). The right coronary artery was compressed by the external aortic right coronary sinus aneurysm (Panel **B**, arrow)

**Fig. 2:**
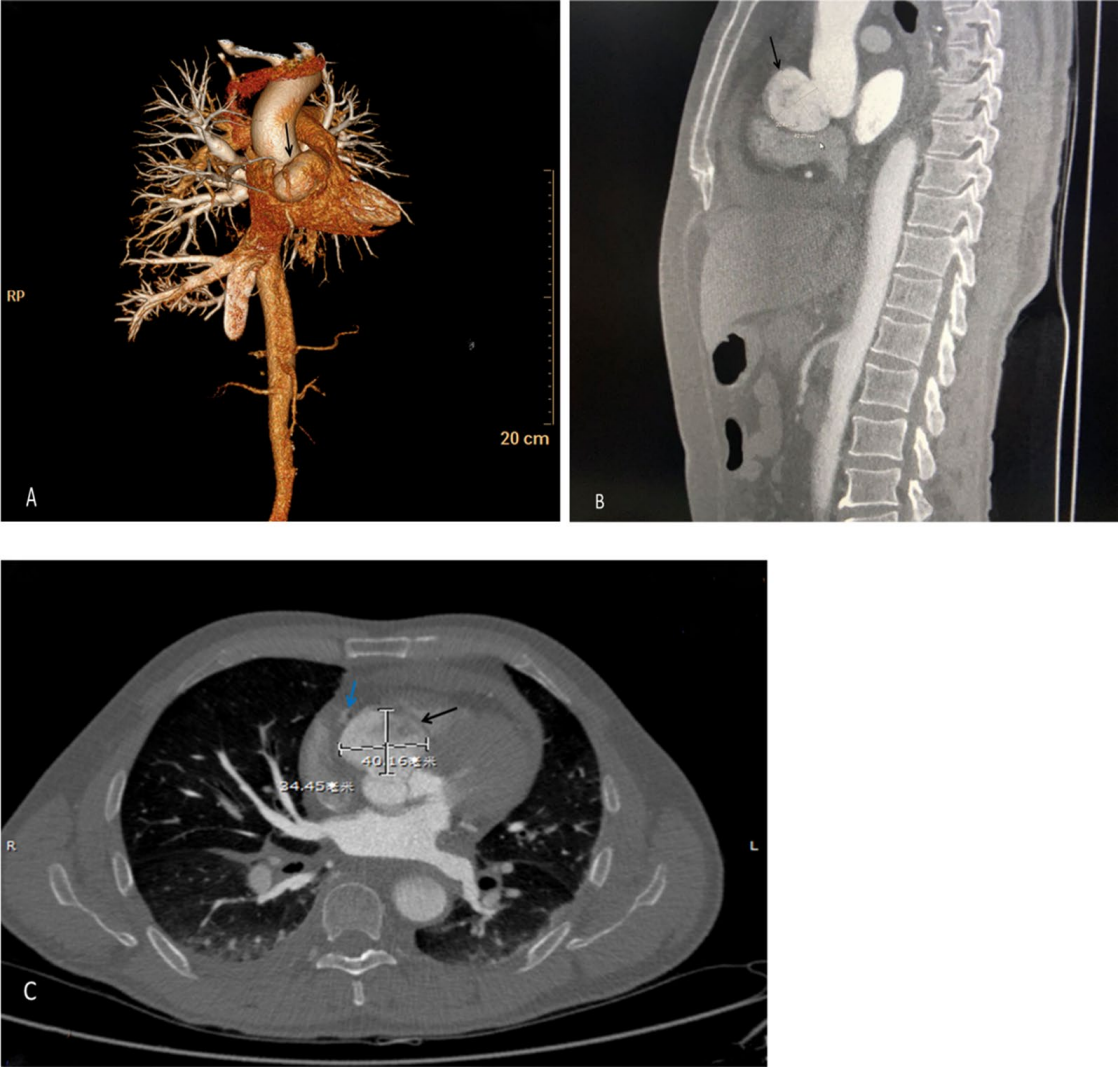
Aorta computed tomography angiography. Aorta computed tomography angiography showed a giant outward aortic right coronary sinus aneurysm (Panel **A**, **B** and **C**, black arrows) that was compressing on the ostium of the RCA (Panel **C**, blue arrow)

**Fig. 3:**
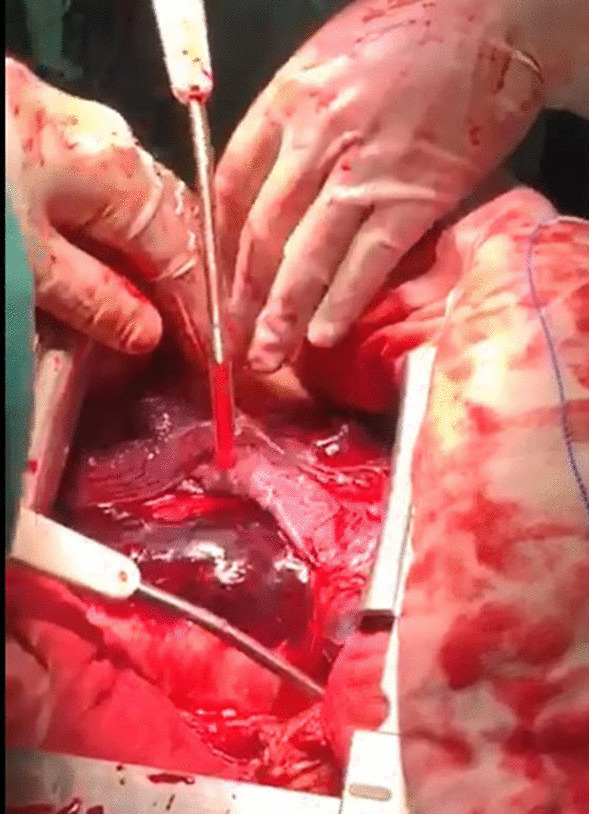
Intraoperative image. A giant hematoma was seen in the aortic root. Surgery confirmed that the hematoma was originating from the aortic right coronary sinus, thereby causing external compression of the right coronary artery. Ruptured SVA protruded into the space between the right atrium and the visceral pericardium

**Fig. 4:**
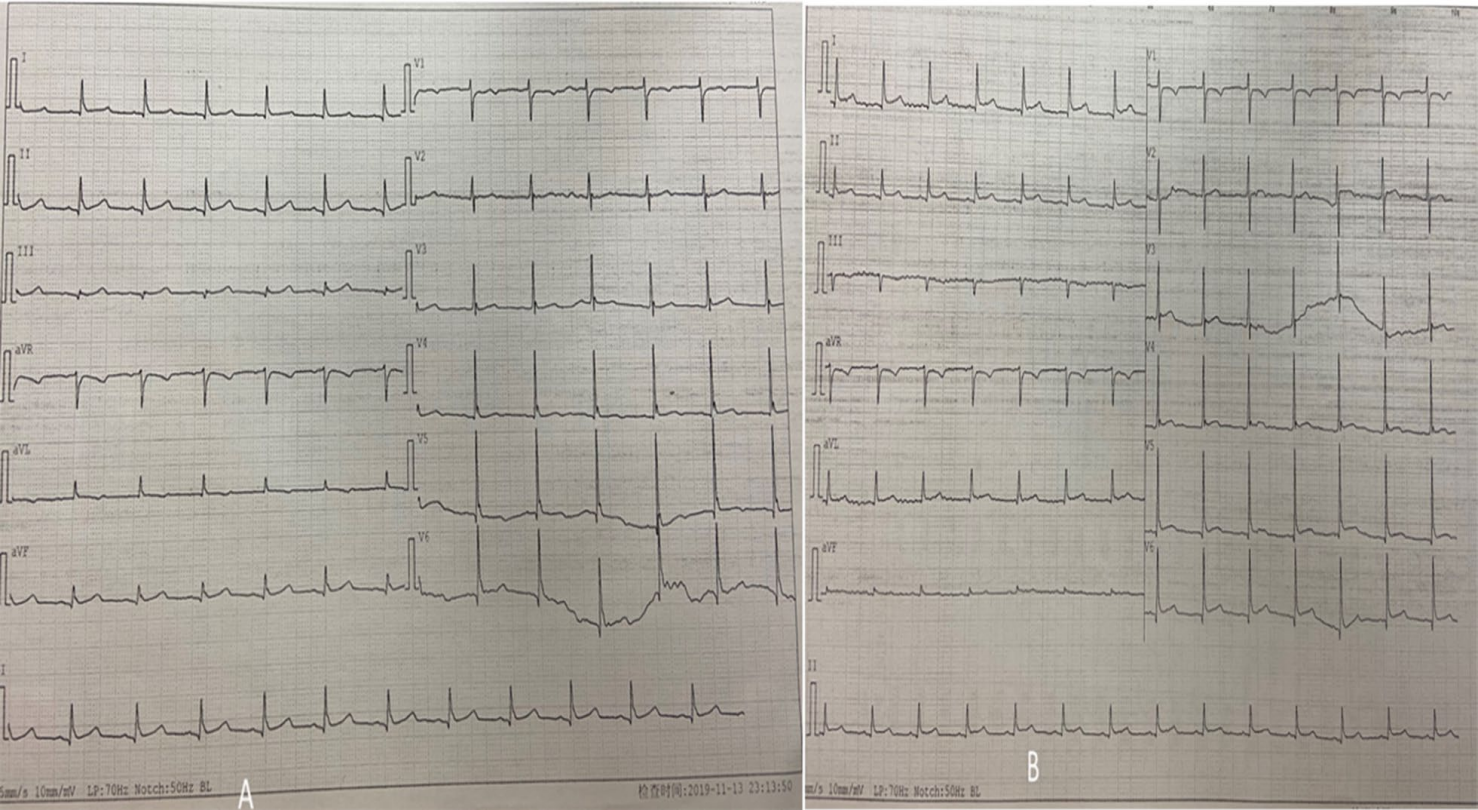
Electrocardiogram. Electrocardiograms obtained on the evening of surgery (**A**) and day 2 after surgery (**B**). 12-lead electrocardiography showed sinus rhythm. **A**: T-wave flat in I, aVL and V_1_ through V_6_ leads, q waves visible in II, III and aVF leads, ST-segment slight elevation in II, III and aVF leads. **B**: ST-segment horizontal elevation in II, III, aVF leads and V5 through V6 leads

Emergency transthoracic echocardiography was performed to evaluate cardiac function, which revealed a left ventricular ejection fraction (LVEF) of 60%, and a small volume of pericardial effusion with an 8 mm liquid dark area in the apex of the heart, 8 mm in left ventricular lateral wall, and 5 mm in right ventricular lateral wall. No major myocardial wall motion abnormalities were seen at the initial evaluation by the emergency physician. Bedside color Doppler ultrasound imaging of abdomen and urinary bladder was normal. After normal saline and norepinephrine were administered, the patient’ condition was relatively stable. Then he was transferred for percutaneous coronary intervention for suspected acute coronary syndrome. Emergency coronary angiography (Fig. 1) showed severe proximal RCA stenosis, which was related to the external compression due to a large mass (30 mm × 45 mm). It also showed severe cardiac hypokinesia, possibly caused by circumferential pericardial effusion. The patient was immediately transferred for complete aorta CTA to achieve an accurate and rapid diagnosis, and for guiding surgery. The aorta CTA revealed the presence of a giant outward aneurysm (40 mm × 34 mm; Fig. 2) of aortic root, which was compressing the ostium of the RCA, as well as moderate pericardial effusion. The patient was rushed to the cardiac surgery unit. The intra-operative findings included moderate hemorrhagic pericardial effusion (about 400 ml), massive blood clot on the right atrioventricular surface, ruptured right SVA, ruptured ostium of RCA, hematoma on the right atrial side and medial pulmonary artery (Fig. 3). Ruptured right SVA repair and RCA reconstruction were successfully performed. Finally, the patient was discharged with no residual symptoms. The ECG after surgery is shown in Fig. 4.

## Discussion and conclusion

In this case, the RSOVA protruded into the space between the right atrium and the visceral pericardium, which led to compression of the right proximal coronary artery. Very few such cases have been reported till date. RSOVA can present as formation of shunting, which may rapidly affect the hemodynamic status.

A prompt differential diagnosis between acute coronary syndrome (ACS) and acute aortic disease (AAS) is difficult. Both need rapid diagnosis and decision-making to reduce the extremely poor prognosis. AAS includes aortic dissection, pseudoaneurysm, aortic rupture, traumatic aortic injury [4]. In this case, fainting was the initial symptom, and upper abdominal pain developed after arriving at the emergency department. The most prominent feature of the patient’s presentation was cardiogenic shock. However, elevated troponin level was discordant with the extent of ventricular dysfunction based on the emergency coronary angiography, so the most likely diagnosis was AAS.

Transthoracic echocardiography is the first-line diagnostic tool for aneurysm, because it can clearly visualize the aneurysm walls and the disturbed blood flow at the site of perforation [5]. However, the sensitivity and accuracy of echocardiography is limited, especially in an emergency (6). CTA can provide additional 2-D or 3-D anatomical information due to its high resolution, which can play an important role in achieving an accurate and rapid diagnosis, and for guiding surgery. In this case, the primary impression was coronary artery disease. In addition, SOVA in the patient was not recognized by transthoracic echocardiography in the emergency department due to the absence of structural anomalies and shunt locations. We hypothesize that the laceration of the aortic sinus may be too small to be detected by emergency beside transthoracic echocardiography. As the disease progresses, emergency coronary angiography found severe stenosis of the proximal RCA resulting from a massive external compression. Next, aortic CTA confirmed the presence of a giant outward aneurysm of aortic root, which was compressing on the ostium of the RCA, and provided excellent anatomical guidance for the surgery. Finally, the patient underwent emergency excision of the right coronary sinus aneurysm, patch repair, and pericardial effusion drainage. The patient recovered uneventfully, and was discharged on postoperative day 20. At his follow-up visit one year later, he had been hemodynamically stable, without any discomfort.

This case highlights the importance of being vigilant about the existence of SOVA. RSOVA should be suspected in a patient presenting with acute hemodynamic compromise, and echocardiography should be immediately performed. Definitive diagnosis often requires cardiac catheterization, and an aortogram should be performed unless endocarditis is suspected.

## Data Availability

Not applicable.

## Abbreviations

SOVA: Sinus of valsalva aneurysm
RSOVA: Ruptured sinus of valsalva aneurysm
RCA: Right coronary artery
CTA: Computed tomography angiography
ECG: Electrocardiogram
hs-cTn: High-sensitivity cardiac troponin
LVEF: Left ventricular ejection fraction
ACS: Acute coronary syndrome
AAS: Acute aortic disease
